# Metabolomic Analyses of Plasma Reveals New Insights into Asphyxia and Resuscitation in Pigs

**DOI:** 10.1371/journal.pone.0009606

**Published:** 2010-03-09

**Authors:** Rønnaug Solberg, David Enot, Hans-Peter Deigner, Therese Koal, Sabine Scholl-Bürgi, Ola D. Saugstad, Matthias Keller

**Affiliations:** 1 Department of Pediatric Research, Oslo University Hospital, Rikshospitalet, University of Oslo, Norway; 2 Department for Surgical Research, Oslo University Hospital, Rikshospitalet, University of Oslo, Norway; 3 BIOCRATES Life Sciences AG, Innsbruck, Austria; 4 Innsbruck Medical University, Department of Pediatrics IV, Division of Neuropediatrics and Inherited Metabolic Disorders, Innsbruck, Austria; 5 Department of Pediatrics I, Neonatology, University Essen, Essen, Germany; Oregon Health and Science University, United States of America

## Abstract

**Background:**

Currently, a limited range of biochemical tests for hypoxia are in clinical use. Early diagnostic and functional biomarkers that mirror cellular metabolism and recovery during resuscitation are lacking. We hypothesized that the quantification of metabolites after hypoxia and resuscitation would enable the detection of markers of hypoxia as well as markers enabling the monitoring and evaluation of resuscitation strategies.

**Methods and Findings:**

Hypoxemia of different durations was induced in newborn piglets before randomization for resuscitation with 21% or 100% oxygen for 15 min or prolonged hyperoxia. Metabolites were measured in plasma taken before and after hypoxia as well as after resuscitation. Lactate, pH and base deficit did not correlate with the duration of hypoxia. In contrast to these, we detected the ratios of alanine to branched chained amino acids (Ala/BCAA; R^2^.adj = 0.58, q-value<0.001) and of glycine to BCAA (Gly/BCAA; R^2^.adj = 0.45, q-value<0.005), which were highly correlated with the duration of hypoxia. Combinations of metabolites and ratios increased the correlation to R^2^adjust = 0.92. Reoxygenation with 100% oxygen delayed cellular metabolic recovery. Reoxygenation with different concentrations of oxygen reduced lactate levels to a similar extent. In contrast, metabolites of the Krebs cycle (which is directly linked to mitochondrial function) including alpha keto-glutarate, succinate and fumarate were significantly reduced at different rates depending on the resuscitation, showing a delay in recovery in the 100% reoxygenation groups. Additional metabolites showing different responses to reoxygenation include oxysterols and acylcarnitines (n = 8–11, q<0.001).

**Conclusions:**

This study provides a novel strategy and set of biomarkers. It provides biochemical *in vivo* data that resuscitation with 100% oxygen delays cellular recovery. In addition, the oxysterol increase raises concerns about the safety of 100% O_2_ resuscitation. Our biomarkers can be used in a broad clinical setting for evaluation or the prediction of damage in conditions associated with low tissue oxygenation in both infancy and adulthood. These findings have to be validated in human trials.

## Introduction

Approximately 5–10% of newborns require some kind of assistance to start breathing after birth [1], and approximately 1% need more extensive interventions. Recent research has shown that the use of extra oxygen for newborn resuscitation negatively influences both morbidity and mortality [2]–[7] However, it is still widely used, and the pathophysiology is not yet fully understood. Furthermore, perinatal asphyxia is a worldwide problem and a leading cause of morbidity as well as mortality in the neonatal period, causing around one million deaths each year [8]. Despite declining incidence of hypoxic-ischemic encephalopathy (HIE) following asphyxia [9], still approximately 1/1000 newborns, and in developing countries even 5–10/1000, suffer from moderate or severe HIE, [10] with at least a 25% risk of permanent neurological impairment [11]–[13]. Therapy has been limited to preventative measures and supportive strategies. Recent experimental and clinical studies clearly show the benefit of induced hypothermia as a clinically feasible maneuver that improves the outcome of neonates with HIE [14], [15]. It has been shown that a delay of hypothermia reduces the neuroprotective potential: the earlier the therapy is initiated, the higher the protective effect [16]. Thus, early biomarkers indicating the duration/severity of hypoxia and enabling risk stratification immediately after asphyxia might be particular beneficial. Such biomarkers indicating the duration/severity of hypoxia seem to be of particular importance in cases of asphyxiated and resuscitated newborns who do not suffer from conventionally diagnosed and defined HIE as these newborns have also been described as having an increased risk of low IQ score at eight years [17]. This makes the search for new biomarkers even more important towards an early start of neuroprotective therapy.

In addition to early biomarkers for the duration of hypoxia, there is also a need for *in vivo* markers of cellular recovery to enable the monitoring and evaluation of resuscitation strategies. Direct functional indicators of cellular metabolic response are currently lacking or of limited use.

The comprehensive quantitative assessment of plasma metabolites might bridge this information gap by depicting such functional information, as metabolite differences in plasma provide the closest link to cellular metabolism in the whole body and its response to asphyxia and resuscitation. Thus, the extensive characterization of the largest possible number of metabolites from relevant or potentially impacted metabolic pathways is a promising approach.

**Figure 1:**
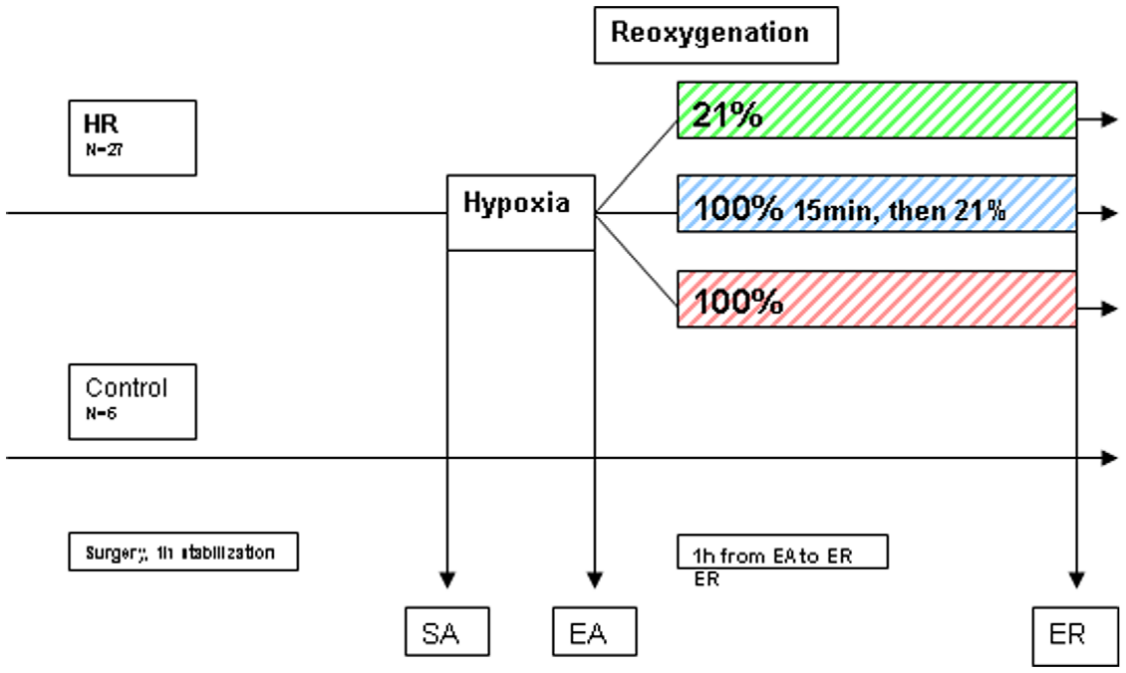
Experimental study design. After one hour of stabilization, 27 piglets were randomized to hypoxia and reoxygenation (HR). Hypoxemia (start of asphyxia, SA) was achieved by ventilation with a gas mixture of 8% O_2_ in N_2_ until either the mean arterial blood pressure decreased to 20 mmHg or the base excess (BE) reached −20 mM (end of asphyxia, EA). Before the start of resuscitation, the hypoxic piglets were block-randomized for resuscitation with 21% or 100% oxygen for 15 min and then ventilation with room air for 45 min (Groups 1 (n = 8) and 2 (n = 8)) or for receiving 100% oxygen for 60 min (Group 3 (n = 11)). Control animals were handled as the other groups without exposure to hypoxia or hyperoxia. Blood samples for metabolomic analyses were taken at the start of hypoxia (SA), the end of hypoxia (EA) and the end of reoxygenation (ER).

The aim of the current study was to evaluate the effects of asphyxia and resuscitation with different oxygen concentrations on the plasma metabolites in newborn piglets. We hypothesized that the comprehensive quantification of plasma metabolites would: 1) enable the identification of a biochemical marker or marker combinations that correlate(s) with the duration of hypoxia better than lactate, pH and base excess; and 2) be a tool to monitor and describe the effects of the therapeutic intervention on a functional level and thus provide biochemical evidence for the cellular integrity as well as metabolism recovery due to different resuscitation protocols. To test these hypotheses, we chose a well-known animal model of perinatal asphyxia. Hypoxemia of different durations was induced in newborn piglets before randomization for resuscitation with 21% or 100% oxygen for 15 min and then 21% oxygen for 45 min or 100% oxygen for 60 min. Quantification of metabolites was carried out on blood samples taken before and after hypoxia and after resuscitation. The experimental study design is visualized in Figure 1.

## Results

### Cohort Characterization during the Experiment

The summary of the cohort characteristics before hypoxia, after hypoxia and after reoxygenation is given in Table 1. The preliminary inspection of the design structure did not reveal significant differences between the treatment groups (Tukey adjusted p value greater than 0.1 for any group comparison) with respect to the clinical parameters (Hb, age, bodyweight, pH, BE, lactate, glucose and MABP) measured after each piglet underwent a one-hour stabilization/relaxation procedure. Hypoxia lasted on average 69±24 minutes (n = 27) and was not found to be significantly different between the three groups (F(2,13.7) = 1.9, p = 0.18). Hypoxia induced significant changes in the pH, lactate, BE and MABP to a similar extent in the three groups that underwent hypoxia (Tukey adjusted p-value<0.001 for any comparison involving the control group and p>0.2 otherwise).

**Table 1:** Characterization of the study cohort.

	21%	100% for 15 min, 21% for 45 min	100% for 60 min	Control	p values
Weight (g)	1948 (134)	1693 (552)	1951 (132)	1868 (78)	0.20
Age (h)	23.0 (5.7)	20.8 (5.1)	21.1 (4.4)	30.0 (2.9)	0.08
Gender M/F	4/4	3/5	5/6	3/3	0.9
Hb g/100 ml start	8.1 (0.9)	7.5 (1.8)	7.5 (1.4)	7.6 (1.3)	0.74
Hypoxia (min)	57 (13)	86 (33)	61 (13)	-	0.06
**pH** start	7.44 (0.005)	7.48 (0.006)	7.44 (0.005)	7.42 (0.007)	0.34
End hypoxia	6.90 (0.04)	6.91(0.05)	6.95 (0.09)	*7.43(0.03)*	<0.001
end reoxygenation	7.34 (0.06)	7.34 (0.07)	7.36 (0.09)	*7.43(0.03)*	0.01
**BE** mmol/L start	3.3 (3.1)	5.4 (3.7)	5.2 (4.7)	1.9 (3.1)	0.26
End hypoxia	−20.2 (2.1)	−19.9 (2.1)	−18.7 (3.7)	*2.64*	<0.001
end reoxygenation	−6.1 (2.5)	−8.0 (4.1)	−3.8 (4.6)	*1.5 (1.4)*	<0.001
**MABP** mmHg start	75 (9)	72 (15)	71 (13)	74 (10)	0.84
End hypoxia	42 (16)	43 (16)	41 (16)	*84 (12)*	<0.001
end reoxygenation	53 (4)	48 (8)	53 (8)	*74 (9)*	<0.001
**SaO_2_**% start	96 (1)	97 (2)	96 (1)	95 (3)	0.53
End hypoxia	29 (3)	30 (5)	30 (5)	*96 (2)*	<0.001
end reoxygenation	94 (2)	95 (2)	100 (0)	*94 (3)*	
**Heart Rate** start	189 (30)	176 (54)	194 (56)	150 (31)	0.13
End hypoxia	216 (24)	230 (20)	206 (43)	*171 (48)*	0.10
end reoxygenation	229 (27)	215 (51)	230 (28)	*167 (39)*	0.03
**pO_2_** kPa start	11.5 (0.6)	11.8 (1.5)	11.3 (0.9)	10.7 (1.8)	0.60
End hypoxia	4.7 (0.4)	4.4 (0.5)	4.5 (0.7)	*11.8 (1.6)*	<0.001
end reoxygenation	11.5 (1.3)	11.9 (2.0)	59.8 (9.1)	*10.6 (2.0)*	<0.001
**pCO_2_** kPa start	5.3 (0.4)	5.2 (0.5)	5.8 (0.5)	5.6 (0.9)	0.14
End hypoxia	8.8 (0.3)	8.7 (0.5)	8.3 (0.8)	*5.5 (1.0)*	<0.001
end reoxygenation	4.9 (0.4)	4.6 (0.79	5.2 (0.7)	*5.3 (0.8)*	0.36
**Glucose** start	6.9 (0.8)	6.6 (1.4)	6.4 (1.8)	7.0 (0.6)	0.78
End hypoxia	10.4 (4.2)	8.0 (3.9)	7.0 (4.5)	*6.2 (0.6)*	0.09
end reoxygenation	8.8 (2.8)	6.9 (2.7)	6.1 (2.6)	*6.1 (0.4)*	0.18
**Lactate** start	2.0 (0.8)	3.2 (2.4)	2.8 (1.0)	2.5 (0.9)	0.38
End hypoxia	13.7 (4.7)	13.6 (4.5)	12.6 (5.4)	*1.5 (0.7)*	<0.001
end reoxygenation	8.6 (2.2)	10.5 (3.3)	8.9 (2.5)|	*1.3 (0.7)*	<0.001

*Characterization of the study cohort before, directly after asphyxia and after reoxygenation, using classically used parameters in the clinic*: The preliminary inspection of the design structure did not reveal significant differences between the treatment groups (Tukey adjusted p value greater than 0.1 for any group comparison) with respect to the clinical parameters (Hb, age, bodyweight, pH, BE, lactate and MABP) measured after the piglets underwent a one-hour stabilization/relaxation procedure. Hypoxia was not found to be significantly different between the three groups (F (2, 13.7)  = 1.9, p = 0.18). Hypoxia induced significant changes in pH, lactate, BE and MABP to a similar extent in the three groups of animals that underwent hypoxia (Tukey adjusted p value<0.001 for any comparison involving the control group and p>0.2 otherwise, n =  8, 8, 11 and 6 for the 21%, 100% for 15 min, 100% for 60 min and control groups, respectively; italic values show the control group at corresponding time points).

### Effect of Hypoxia and Reoxygenation on Plasma Metabolites Is Independent of the Mode of Reoxygenation

**Figure 2:**
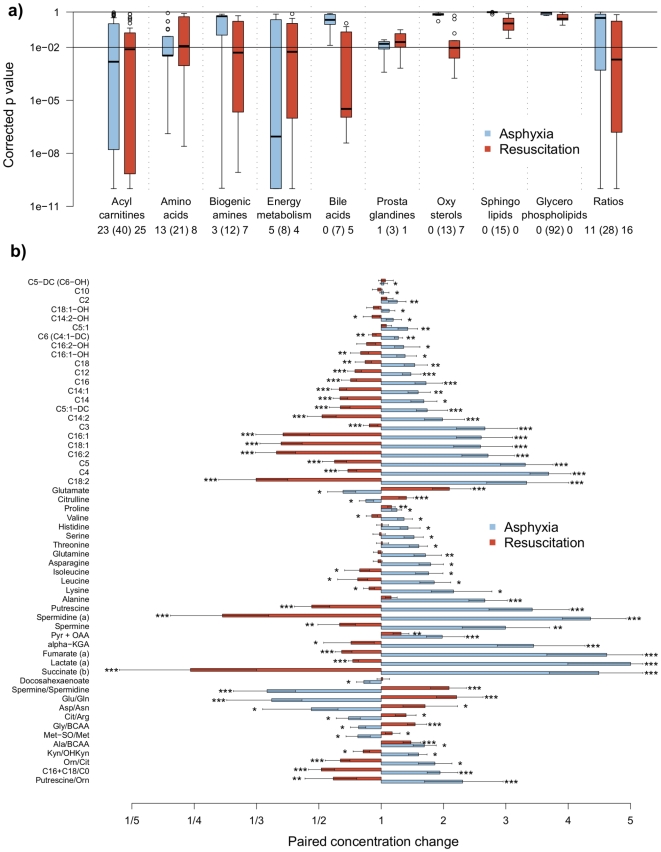
Distribution of the metabolites classes. The distribution of the corrected p value according to the metabolites classes from the two linear models describing alterations between control and treated animals during asphyxia (left boxplot) and changes in the plasma metabolome during reoxygenation (right boxplot). The number of significant changes at q-value<0.01 is appearing below each boxplot, alongside with the total number of metabolites for a given class in brackets. A detailed description of each group of metabolites is given in Table S1. Figure 3b) Concentration changes of metabolites for treated animals during asphyxia (EA/SA, blue) and during reoxygenation (ER/EA, orange). Levels of significance are as follows: *, q<10-2, **, q<10-4 and *** q<10-6.

**Figure 3:**
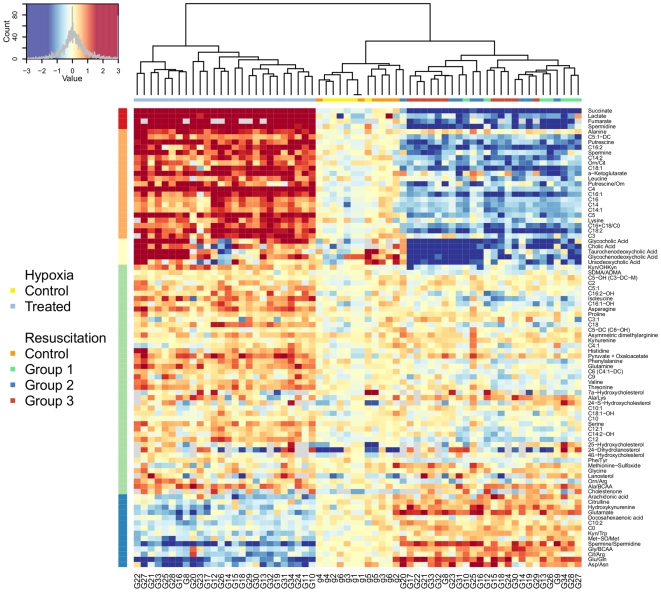
Visualization of individual metabolite concentration ratios. The heat-map is a graphical representation of the true metabolite concentration changes in a two-dimensional, rectangular and colored grid. Metabolites are given on the y axis (i.e., row), animal profiles are given on the x axis (i.e., columns) and each “pixel” represents a metabolite change (in log basis 2 scale) between two consecutive time points. With regard to the design, each animal (as labeled by G/g) is thus represented twice: one cell for each change corresponding to the hypoxia (i.e., EA/SA) and to the resuscitation (i.e., ER/EA) steps. For a more comprehensive visualization, metabolite changes are centered around a common value, and both rows and columns are reordered so that meaningful characteristics of the data can be uncovered without any a priori knowledge about the experimental design. First, concentration changes for each metabolite (row) are centered around the average change found in the sham animals (i.e., control animals labeled by g). Following the left corner diagram, red (resp. blue)-colored cells indicate a higher (resp. a lower) change in concentration than the average change observed for the control animals. Cells for which changes are greater than 3 (c.a. 2∧3 = 8 fold change) and lower than −3 (c.a. 1/8) are coded in the darkest red and blue. Column reordering proceeds by placing observations (i.e., EA/SA or ER/EA from one animal) with the most similar profiles, using hierarchical clustering agglomeration in which the leaves represent individual observations and in which the height of the nodes reflects the dissimilarity between the two clusters of observation. Animal labels are given at the bottom, whereas a square color-coded according to its treatment group is at the top of the heat-map. Three clusters, corresponding to the profiles from the asphyxiated animals (left) followed by the sham animals (center) and profiles associated to resuscitation (right), are clearly observed. However, unsupervised clustering cannot efficiently group animals according to their reoxygenation protocol (right). Finally, metabolites (i.e., rows) are grouped according to their intensity patterns, with their partitions displayed on the left side. The upper part of the heat-map comprises metabolites that are increased during asphyxia and decreased during resuscitation, whereas the lower part regroups compounds with the opposite behavior.

The effect of hypoxia on plasma metabolites was assessed by metabolite concentration changes before and after hypoxia between asphyxiated animals and control animals. Out of 213 metabolites that could be quantified, 45 analytes and 11 ratios were found to be significant at p-values<0.01. Figure 2a illustrates the corrected p-value distribution due to the effect of asphyxia. Predominant changes due to asphyxia include metabolites belonging to acylcarnitines, amino acids, biogenic amines and members of the energy metabolism pathway, whereas lipids, bile acids and oxysterols were not disrupted directly after asphyxia. The investigation of chemical changes associated with the reoxygenation, independent of the mode of reoxygenation, lead to similar conclusions where 57 compounds and 16 ratios were significantly changing at p-values<0.01. Figure 2b provides a detailed presentation of the effect of asphyxia as well as the resuscitation on the degree of metabolite changes due to asphyxia and resuscitation (data are given as mean fold change of metabolite concentration during asphyxia (blue) and during reoxygenation (orange); *q<10^−2^, ** q<10^−4^ and *** q<10^−6^ versus controls at same time points). To illustrate the individual changes of metabolite levels due to the hypoxia challenge as well as reoxygenation in each animal, the heat-map in Figure 3 is provided. In this heat-map, each metabolite (right y- axis) that changed significantly in each animal (x-axis) is provided. Each animal is thereby presented twice: first for the metabolite change due to hypoxia, and secondly for the metabolite change due to reoxygenation (compared to the controls for the corresponding time interval). The color reflects the direction of change (blue: decrease, red: increase), while the color intensity reflects the level of significance. The grouping of animals (x-axis) is a result of an unsupervised clustering of the animals according to their metabolite pattern. Three groups (clusters), corresponding to the profiles from the hypoxic animals (left) followed by the sham animals (center) and profiles associated to reoxygenation (right), are clearly observed. Even under standardized conditions, each individual animal has its individual metabolomic response due to hypoxia. In the control animals, there were no significant changes of the metabolites. In the reoxygenated animals, although there is a clear clustering of the animals, unsupervised clustering did not efficiently group animals according to their reoxygenation protocol (right).

### Correlation with the Duration of Hypoxia

**Figure 4:**
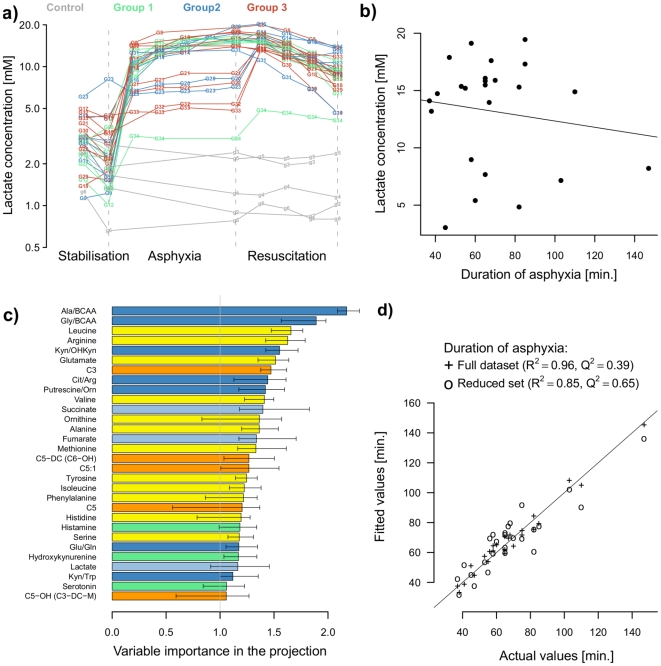
Correlation of metabolite concentration with the duration of hypoxia. The time-course of plasma lactate levels (in mM) of all individual animals before, during and after asphyxia in a normalized discrete timescale (animals are sampled at 15/30 minutes intervals during asphyxia and resuscitation/reoxygenation) (a). Lactate concentration (mM) does not correlate with the duration of hypoxia (in minutes) (b). The fitted values of the PLS models built on the full set (+) or reduced (*) set of metabolites and ratios of metabolites are plotted against the actual duration of asphyxia (in minutes) Combinations of metabolites by PLS modeling provided a better representation of the duration of asphyxia (c). n = 8–11. The 30 most contributing features are sorted according to the variable importance on the projection (VIP) scores; VIP are given as median and 20/80 quantiles from 30 resampling steps (d).

Figure 4a shows the tracing of lactate levels and its change due to hypoxia and resuscitation in each animal. In most but not all of the animals, hypoxia resulted in an immediate increase of lactate within the first 15 minutes. Thereafter, the lactate levels remained stable in most of the animals. The correlation of lactate levels with the duration of hypoxia revealed no correlation, as shown in Figure 4b (R^2^.adj = 0.13, p = 0.36). Similarly, base excess (R^2^.adj = 0.01, p = 0.4) and pH (R^2^.adj = 0.03, p = 0.5) also did not correlate with the duration of hypoxia. In contrast, we detected the ratios of alanine to branched chained amino acids, Ala/BCAA (R^2^.adj = 0.58, q-value<0.001), and of glycine to BCAA, Gly/BCAA (R^2^.adj = 0.45, q-value<0.005), which per se highly correlated with the duration of hypoxia. Figure 4d shows the results of multivariate modeling of the duration of hypoxia by mean of partial least square (PLS) including acylcarnitines, amino acids, intermediates of energy metabolism, biogenic amines and any ratios involving these compounds. The combination of different metabolites and metabolite ratios results in a strong correlation with the duration of hypoxia. Figure 4c highlights the variables showing the 30 largest VIP (Variable Importance in the Projection) values that were extracted from the PLS model, shedding light onto the role played by specific metabolites and ratios in modeling the length of hypoxia. After reducing the dataset to metabolites and ratios with VIP values greater than 1, the predictive power of the PLS model was increased: R^2^ = 0.85, Q^2^ = 0.65, RMSE = 13.9 minutes, p<0.0001 (Figure 4d).

### Effect of Resuscitation Protocols on Plasma Metabolites

**Figure 5:**
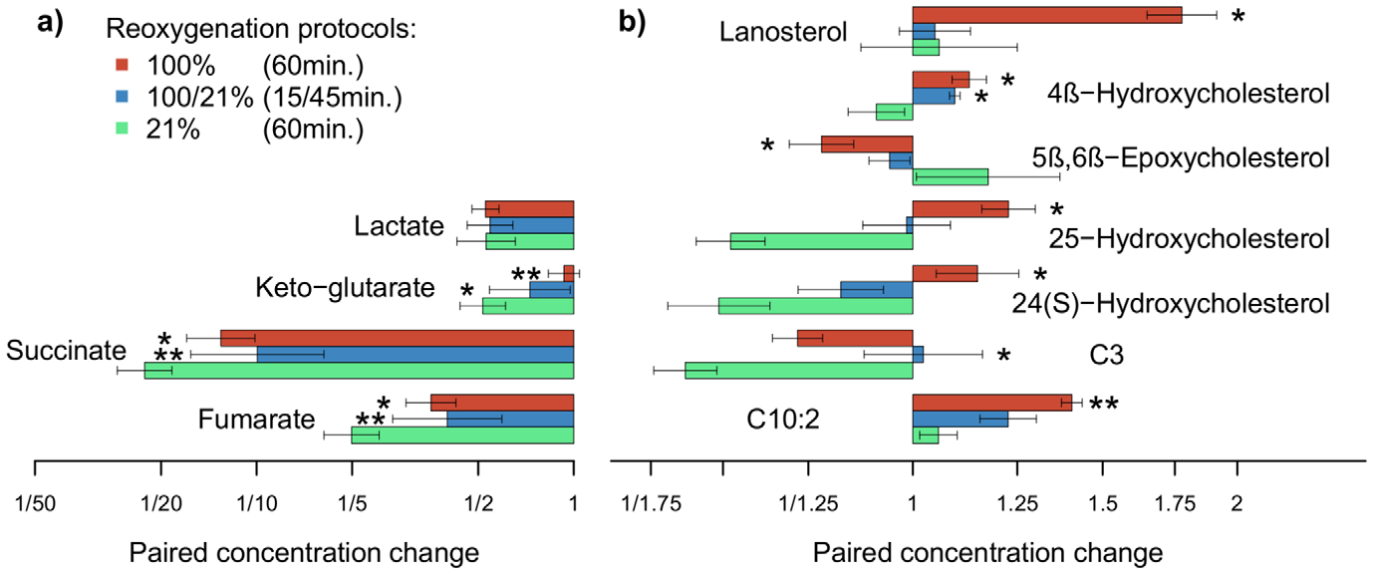
Concentration ratios of metabolites due to resuscitation protocols. An explicit summary of concentration ratios for the set of metabolites found significant at a q value<0.01 due to resuscitation protocols (n = 8–11). Bars correspond to the paired changes during reoxygenation (to the left–decline; to the right–increase). Bars in: green: resuscitation with 21%O_2_; blue: 100% O_2_ for 15 min and 45 min of 21% O_2_; and red: prolonged hyperoxia, 100% for 60 minutes. Levels of significance are coded as follows: *, q<10^−2^, **, q<10^−4^.

Figures 5a and 5b illustrate metabolites that are significantly affected by the different modes of reoxygenation. Four small acids exhibited dramatic increases during hypoxia (resp. 850%, 266%, 8000% and 587% for lactate, alpha keto-glutarate, succinate and fumarate, respectively). Reoxygenation led to a decrease of these metabolites, but whereas lactate levels declined in all three groups to a similar extent, the Krebs cycle intermediates alpha keto-glutarate, succinate and fumarate were significantly reduced at different rates depending on the resuscitation protocol (Figure 5a), showing a faster decline in the 21% reoxygenation group. Additional metabolites showing different responses to reoxygenation included oxysterols and acylcarnitines (Figure 5b) (data are given as mean fold changes during reoxygenation + SEM. Levels of significance in Figures 5a–b are coded as follows: *, q<10^−2^, **, q<10^−3^ vs. group 1).

## Discussion

In this study, we performed a comprehensive quantitative characterization of plasma metabolites in newborn piglets before and after hypoxia as well as after reoxygenation with different oxygen concentrations. We describe two major findings:

First, hypoxia induced significant changes in plasma metabolites. Interestingly, clinical used parameters like lactate, base deficit and pH did not correlate with the duration of hypoxia. In contrast, we identified (to the best of our knowledge) for the first time a set of markers with good correlation to the duration of hypoxia.

Second, reoxygenation also induced a broad change of plasma metabolites, with significant differences depending on the resuscitation protocol. The pronounced decline of the Krebs cycle intermediates succinate, fumarate and alpha keto-glutarate indicates an earlier recovery of mitochondrial function when 21% of oxygen is used for resuscitation compared to 100% oxygen.

Therefore, these findings may have major importance for i) the diagnosis of asphyxia not only in newborns and ii) the treatment of asphyxiated newborn infants.

### Effect of Asphyxia on Plasma Metabolites

Exposure to severe, prolonged hypoxia revealed changes in metabolites belonging to acylcarnitines, amino acids, biogenic amines and members of the energy metabolism pathway. Thereby we detected decreases of free carnitine (C0) and decadienyl-L-carnitine (C10∶2) and an increase in mainly long chain acyl carnitines after hypoxia. This is in agreement with other data, where levels of free and total carnitine were lower and levels of long chain acyl carnitines were higher in asphyxiated newborn babies than in controls [18], [19]. It was speculated that they may serve as fuel for cerebral energy metabolism [20]. Our results show that incomplete fatty acid oxidation during hypoxia leads to an increase of coenzyme A esters of fatty acids, which are bound to carnitine forming acylcarnitines and therefore contribute to the detoxification of the free fatty acids, which have been shown to have toxic potential. The decrease in carnitine is of concern, as carnitine is required for a normal mitochondrial function [21], [22]. In addition, newborns have an increased risk of cellular injury related to carnitine deficiency because of the immaturity of the carnitine biosynthetic pathways [23], [24]. L-carnitine can suppress lipid peroxydation after ischemia reperfusion [25] and can suppress hydroxyl radical production in the Fenton reaction. Together with propionyl-L-carnitine (C3), L-carnitine possesses potent antioxidant activity [26]. The lack of carnitine may thereby give more oxidative stress. Experimentally, L-carnitine treatment after HI has been associated with a reduction in neurological injury [27]. An antiapoptotic role of carnitine has also been proposed [28], [29]. Because L-carnitine possesses minimal toxicity and has been in extensive use in pediatric clinical settings, it is an attractive drug for perinatal asphyxia [30]. However, carnitine supplementation is still controversial. It has been widely discussed whether supplementation of exogenous carnitine is advisable for the recovery of reduced intracellular carnitine concentrations [31], [32]. In addition to the acylcarnitine data, the results obtained in this study go far beyond the current knowledge as the investigation reveals additional metabolites and metabolite ratios that describe the status of asphyxia on a cellular level in much greater detail than acylcarnitines alone. Currently, we cannot explain every single finding, and at this stage, we can only speculate about the biochemical pathways involved. This is far beyond the aim of the study and the possibilities given in one manuscript. Additional analyses are required, and we share all findings to open this up to other investigators. However, the comprehensive analyses of plasma metabolites enabled us to investigate whether other metabolites, ratios and/or combinations of metabolites do correlate with the duration of hypoxia and to compare these findings with the correlations of clinically used parameters.

### Correlation with the Duration of Hypoxia

The analysis of metabolite changes during hypoxia in relation to the duration of hypoxia revealed components that could explain and predict the variation in the time of hypoxia. The ratios of Ala/BCAA and Gly/BCAA alone are highly significant predictors of the duration of hypoxia. By combining these ratios with other metabolites, e.g., intermediates of the Krebs cycle (succinate) and propionyl-L-carnitine (C3), the correlation with the duration of hypoxia was increased to R^2^ = 0.96. The fact that the Ala/BCAA and Gly/BCAA ratios are highly correlated with the duration of hypoxia can be partially explained by the reduction of the metabolic noise by relating the measured concentrations to parameters that are not involved in the investigated pathway but that reflect variations in, e.g., analytical factors and absorption rates [33]. The observation that lactate, pH and base excess do not correlate with the duration of hypoxia (except for the first 15–30 minutes) can be explained by the fact that these parameters are impacted by individual enzymatic activities, nutritional status and compensatory mechanisms. The important roles of succinate and C3 in the statistical model can be seen as the result of their strong direct associations with mitochondrial function, thereby mirroring direct mitochondrial activity. Although we cannot provide an explanation for all of the metabolites contributing to the biomarker set, the detailed characterization of the metabolomic response enables discussion and opens a new field of research. These data led us to propose a new concept of using several metabolites to characterize the severity of hypoxia and thus the risk stratification of tissue injury.

### Effect of Different Reoxygenation Protocols

**Figure 6:**
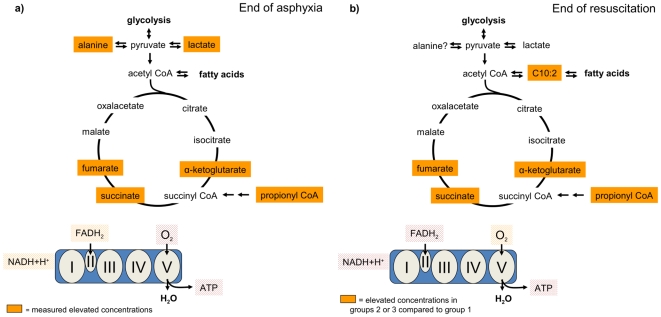
Visualizations into biochemical pathways. Summary and visualizations into biochemical pathways: *a) Effect of asphyxia on Krebs cycle intermediates*; Fumarate, succinate and α-ketoglutarate are strongly increased after asphyxia, as well as lactate and alanine. These increases can be seen as the result of mitochondrial dysfunction and the subsequent disturbance of the Krebs cycle, since the Krebs cycle and mitochondrial function is coupled. *b) Effect of different resuscitation protocols on these intermediates*. Reoxygenation with 100% oxygen for either duration (g2/3) resulted in a significant delay in the decrease in fumarate, succinate and α-ketoglutarate compared to normoxic resuscitation (g1). The significantly lower levels of these Krebs cycle intermediates in the 21% FiO_2_ resuscitation group indicates better mitochondrial recovery.

Fumarate, succinate and alpha keto-glutarate are intermediates of the Krebs cycle (Figure 6) and are strongly increased after asphyxia, as are lactate and alanine. This increase is the result of mitochondrial dysfunction and the subsequent disturbance of the Krebs cycle and accumulation of metabolites involved in upstream biochemical pathways (Figure 6a). The lack of oxygen as an electron acceptor in the respiratory chain results in a diminished production of ATP. Additionally, the NADH+H^+^/NAD^+^ ratio is elevated as these metabolites cannot enter the respiratory chain for energy production and may serve as cofactors for the formation of lactate.

The reoxygenation by the different resuscitation protocols resulted in a decrease of these intermediates, with significant differences between the different groups. Interestingly, reoxygenation with the high oxygen supplies in group 2 and 3 led to a slower decline of Krebs cycle intermediates, indicating a longer lasting disturbance of the Krebs cycle and respiratory chain (Figure 6b). These results are in accordance with other findings of *in vitro* studies showing that hyperoxia causes mitochondrial dysfunction and cell death by an excessive cellular production of reactive oxygen species (ROS) [34]. Hyperoxia results in an uncoupled oxidative phosphorylation in the mitochondrial respiration chain through a change in the expression levels of Cytochrome b, an important component of Complex III, and ATPase 6, 8, important components of enzyme complex V [35]. Thus, the present study provides the first *in vivo* data showing that hyperoxia causes a delayed decrease in metabolites that are biochemically directly linked to mitochondrial function, thereby supporting the *in vitro* data.

In addition to the intermediates of energy metabolism, the lanosterol and oxysterol levels changed depending on the resuscitation protocol. Lanosterol was significantly increased after resuscitation with 100% and prolonged hyperoxia (Group 3). Lanosterol is the common biosynthetic precursor of cholesterol and is converted to cholesterol at the sarcolem. An increase of lanosterol level indicates an inefficient cholesterol synthesis. Cholesterol is an important compound that is incorporated during brain development, and several studies have shown that deficits in cholesterol synthesis [36] or transport [37]–[39] cause neurodegeneration and severe neurological symptoms. Furthermore, the oxysterols 24S-Hydroxycholesterol (24S-OHC) and 25-Hydroxycholesterol (25-OHC) were significantly increased after prolonged hyperoxia (group 3). 24S-OHC has been used as a biomarker of brain cholesterol balance and as a marker of CNS neuronal mass [40]. Its increase has been associated with acute neuronal damage because it is able to cross the blood-brain barrier (BBB) [41]. Since 24S-OHC has been described as a brain-specific indicator of acute neuronal damage, this, in addition with the increased levels of lanosterol, raises concerns about the exposure of 100% oxygen to asphyxic newborns.

### Clinical Importance

The findings in this study have, in our perspective, major implications for neonatology and neonatal intensive care medicine, as well as for other insults associated with the lack of oxygen and in adulthood.

With respect to neonatology, this study provides new biomarkers that might be useful in a clinical setting for early risk stratification after perinatal asphyxia, enabling an early start of therapeutic interventions like hypothermia. Because we did not assess brain injury per se, we did not detect biomarkers of brain injury per se. However, the study by Odd et al. clearly showed cognitive deficits in cohorts suffering from asphyxia and requiring resuscitation without the classical diagnosis of HIE [17]. There seems to be a gap in diagnosing and evaluating the severity of asphyxia. As we in this study as well as others in clinical trials have shown [42], the conventionally used parameters like pH are of limited use and do not correlate with the neurological outcome. In clinical practice, caregivers are challenged by newborns with low pH and the limited knowledge about the interpretation of this. The metabolites detected in this study that do correlate with the duration of hypoxia might help in the clinical setting for either introducing therapy at an early stage or for risk stratification and close surveillance by other methods like amplitude integrated electroencephalography (aEEG) [42], [43]. Furthermore, the results add new insights for answering the question of the ideal mode of resuscitation for newborns, particularly in the context of other recent findings showing that the use of extra oxygen for newborn resuscitation negatively influences both morbidity and mortality [2]–[7] The effect on Krebs cycle intermediates indicates the earlier recovery of mitochondrial function when 21% oxygen is used for resuscitation.

The same biomarkers and metabolomic approach may also be useful in evaluating or predicting damage in other insults, like after cardiac arrest, traumatic brain injury or conditions of low oxygenation associated with low cardiac output in both infancy and adulthood [20] (LeWcen, Daniel et al: Serum Metabolomic Biomarkers in Hypothermic and Normothermic Models of Hemorrhagic Shock Associated with Increased Mortality, 5^th^ International Conference of the Metabolomics Society, unpublished data).

### Limitations of the Study

As highlighted above, we could not correlate our findings with other technologies like MRI for indicating the severity of brain damage, as the tissue collection was performed for other purposes, e.g., the analyses of neuroprostanes at early timepoints. The resuscitation with 100% for 15 min resulted in increased levels of neuroprostanes, known markers of cerebral injury, in the same animals (data not shown).

Furthermore, this study was performed in a neonatal, not perinatal, model of hypoxia-reoxygenation. However, newborn pigs have a structure and size equivalent to newborn infants as well as have similar immunological and metabolic functions [44]–[46].

### Summary and Conclusion

We identified markers and marker combinations that enable better risk stratifications after asphyxia as well as that indicate cellular recovery better than the conventionally used biochemical parameters (pH, BE and lactate). In addition, we provided *in vivo* data showing that resuscitation with hyperoxia delays cellular recovery. Because metabolites are largely species-independent, our findings offer promising features paving the way to alternative and more detailed diagnostic tools for asphyxia and resuscitation. These biomarkers have to be validated in human trials.

## Materials and Methods

### Approval

The National Animal Research Authority, (NARA), approved the experimental protocol. The animals were cared for and handled in accordance with the European Guidelines for Use of Experimental Animals by certified FELASA fellows (Federation of European Laboratory Animals Science Association).

### Surgical Preparation and Anesthesia

A total of 33 newborn Noroc (LyxLD) pigs were included in the study, with inclusion criteria of 12–36 h, Hb>5 g/dL and good general condition. Twenty-seven of these went through the experimental procedures, and a reference group consisting of six newborn pigs went through the same experimental set-up (were anesthetized, sham operated and ventilated) but were not subjected to hypoxia and reoxygenation (Figure 1).

Anesthesia was induced by giving sevofluran 5% (Sevorane, Abbott); an ear vein was cannulated, and the piglets were given pentobarbital sodium at 15 mg kg^−1^ and fentanyl at 50 µg kg^−1^ intravenously as a bolus injection. The piglets were orally intubated and then placed in the supine position and washed for sterile procedures. Anesthesia was maintained by continuous infusion of fentanyl (50 µg kg^−1^ h^−1^) and midazolam (0.25 mg kg^−1^ h^−1^) in mixtures, giving 1 mL kg^−1^ h^−1^ for each drug applied by IVAC P2000 infusion pump. When necessary, a bolus of fentanyl (10 µg kg^−1^), midazolam (1 mg kg^−1^) or pentobarbital (2.5 mg kg^−1^) was added (need for medication being defined as shivering, trigging on the respirator, increased tone assessed by passive movements of the limbs, increase in blood pressure and/or pulse). A continuous IV infusion (Salidex: saline 0.3% and glucose 3.5%, 10 mL kg^−1^ h^−1^) was given until hypoxia and from 15 min after the start of resuscitation and throughout the experiment. The piglets were ventilated with a pressure-controlled ventilator (Babylog 8000+; Drägerwerk, Lübeck, Germany). Normoventilation (arterial carbon dioxide tension (PaCO_2_) 4.5–5.5 kPa), and a tidal volume of 6–8 mL kg^−1^ were achieved by adjusting the peak inspiratory pressure or ventilatory rate. The ventilatory rate was 15–40 respirations/min. The inspiratory time of 0.4 s and the positive end-expiratory pressure of 4.5 cm H_2_O were kept constant throughout the experiment. The inspired fraction of O_2_ and the end-tidal CO_2_ were continuously monitored (Datex Normocap Oxy; Datex, Helsinki, Finland). The left femoral artery was cannulated with polyethylene catheters (Porex PE-50, inner diameter 0.58 mm; Porex Ltd Hythe, Kent, UK). The mean arterial blood pressure (MABP) was measured continuously in the left femoral artery using BioPac systems MP150-CE. Rectal temperature was maintained between 38.5 and 39.5°C with a heating blanket and a radiant heating lamp. One hour of stabilization was allowed after surgery. At the end of the experiment, the piglets were given an overdose of 150 mg kg^−1^ pentobarbital intravenously.

### Experimental Protocol

Hypoxemia was achieved by ventilation with a gas mixture of 8% O_2_ in N_2_ until either the mean arterial blood pressure decreased to 20 mmHg or the base excess (BE) reached −20 mM. CO_2_ was added during hypoxemia, aiming at a PaCO_2_ of 8.0–9.5 kPa to imitate perinatal asphyxia. Before the start of resuscitation, the hypoxic piglets were block-randomized for resuscitation with 21% or 100% oxygen for 15 min and then ventilation with room air for 45 min (Groups 1 (n = 8) and 2 (n = 8)) or for receiving 100% oxygen for 60 min (Group 3 (n = 11)). After initiating the reoxygenation, the piglets were kept normocapnic (PaCO_2_ 4.5–5.5 kPa). Throughout the whole experiment, there was a continuous surveillance of blood pressure, saturation, pulse, temperature and blood gas measurements. Hemoglobin was measured on a HemoCue Hb 201+ (HemoCue AB, Angelholm, Sweden) at baseline and at the end of the experiment. Temperature-corrected arterial acid/base status and glucose were regularly measured throughout the experiment on a Blood Gas Analyzer 860 (Ciba Corning Diagnostics, Midfield, Mass., USA). Plasma samples for metabolomic analyses were drawn before initiating the hypoxia, at the end of hypoxia and 60 min after initiating reoxygenation and were handled according to standard operating procedures provided by Biocrates Life Sciences AG and then stored at minus 70°C until subsequent analysis. All blood samples obtained from the femoral artery catheter were replaced by normal saline 1.5 x the volume drawn. One hour after the end of hypoxia, the animals were given an overdose of pentobarbital (150 mg kg^−1^ iv). The study staff and the laboratory personnel were blinded to the percentage of oxygen administered by resuscitation.

### Analyses

#### General analyses

Sample preparation and metabolomic analyses were performed at Biocrates life sciences AG, Innsbruck, Austria. We used a multi-parametric, highly robust, sensitive and high-throughput targeted metabolomic platform consisting of flow injection analysis (FIA)-MS/MS and LC-MS/MS methods for the simultaneous quantification of a broad range of endogenous intermediates, namely acylcarnitines, sphingomyelins, hexoses, glycerophospholipids, amino acids, biogenic amines, bile acids, oxysterols and small organic acids, in plasma. A detailed list of all analyzed metabolites is provided in Table S1. All procedures (sample handling, analytics) were performed by co-workers blinded to the experimental groups.

#### Acylcarnitines, sphingomyelins, hexoses, glycerophospholipids (FIA-MS/MS)

To determine the concentration of acylcarnitines, sphingomyelins and glycerophospholipids in plasma, the Absolute*IDQ* kit p150 (Biocrates Life Sciences AG, Innsbruck, Austria) was prepared as described in the manufacturer's protocol. In brief, 10 µL of plasma was added to the center of the filter on the upper 96-well kit plate and was dried using a nitrogen evaporator (VLM Laboratories, Bielefeld, Germany). Subsequently, 20 µL of a 5% solution of phenyl-isothiocyanate was added for derivatization. After incubation, the filter spots were dried again using an evaporator. The metabolites were extracted using 300 µL of a 5 mM ammonium acetate solution in methanol. The extracts were obtained by centrifugation into the lower 96-deep well plate, followed by a dilution step with 600 µL of kit MS running solvent. Mass spectrometric analysis was performed on an API4000 Qtrap® tandem mass spectrometry instrument (Applied Biosystems/MDS Analytical Technologies, Foster City, CA) equipped with an electro-spray ionization (ESI)-source, using the analysis acquisition method as provided in the Absolute*IDQ* kit. The standard FIA-MS/MS method was applied for all measurements with two subsequent 20-µL injections (one for positive and one for negative mode analysis). Multiple reaction monitoring (MRM) detection was used for quantification, applying the spectra parsing algorithm integrated into the MetIQ software (Biocrates Life Sciences AG, Innsbruck, Austria). The concentrations for 148 metabolites (all analytes were determined with the metabolomics kit except for the amino acids, which were determined by a different method) obtained by internal calibration were exported for comprehensive statistical analysis.

#### Amino acids, biogenic amines (LC-MS/MS)

Amino acids and biogenic amines were quantitatively analyzed by reversed phase LC-MS/MS to obtain the chromatographic separation of isobaric (same MRM ion pairs) metabolites for individual quantification performed by external calibration and by use of internal standards. A 10 µL sample volume is required for the analysis using the following sample preparation procedure. Samples were added on filter spots placed in a 96- solvinert well plate (internal standards were placed and dried down under nitrogen before), fixed above a 96 deep well plate (capture plate). 20 µL of 5% phenyl-isothiocyanate derivatization reagent was added. The derivative samples were extracted after incubation by aqueous methanol into the capture plate. Sample extracts were analyzed by LC-ESI-MS/MS in positive MRM detection mode with an API4000 Qtrap® tandem mass spectrometry instrument (Applied Biosystems/MDS Analytical Technologies, Foster City, CA). The analyzed individual metabolite concentrations (Analyst 1.4.2 software, Applied Biosystems, Foster City, CA) were exported for comprehensive statistical analysis.

#### Bile acids (LC-MS/MS)

A highly selective reversed phase LC-MS/MS analysis method in negative MRM detection mode was applied to determine the concentration of bile acids in plasma samples. Samples were extracted via dried filter spot technique in a 96-well plate format, which is well suitable for high-throughput analysis. For highly accurate quantification, internal standards and external calibration were applied. In brief, internal standards and a 20 µL sample volume placed onto the filter spots were extracted and simultaneously protein precipitated with aqueous methanol. These sample extracts were measured by LC-ESI-MS/MS with an API4000 Qtrap® tandem mass spectrometry instrument (Applied Biosystems/MDS Analytical Technologies, Foster City, CA). Data of bile acids were quantified with Analyst 1.4.2 software (Applied Biosystems, Foster City, CA,) and finally exported for comprehensive statistical analysis.

#### Oxysterols (LC-MS/MS)

Oxysterols were quantitatively analyzed by reversed phase LC-ESI-MS/MS to realize liquid chromatographic separation and thus individual quantification of isobaric oxysterols. The most selective detection was performed in positive MRM detection mode using a 4000 Qtrap® tandem mass spectrometry instrument (Applied Biosystems/MDS Analytical Technologies, Foster City, CA). Data were quantified with Analyst 1.4.2 software (Applied Biosystems, Foster City, CA). Ratios of external to internal standards were applied for quantification by means of external 6-point calibration. A sample volume of 20 µL (plasma) was necessary for the analysis. The sample preparation included: I) protein precipitation by placing a 20 µL sample volume on the filter spot, and precipitation by 200 µL Naïve; II) hydrolysis by 100 µL of 0.35 M KOH in 95% ethanol for 2 hrs; III) a washing step (3×200 µL H_2_O) to remove hydrolysis reagent; and, finally, IV) extraction by means of 100 µL aqueous methanol. The 20 µL sample extracts were analyzed by the developed LC-ESI-MS/MS method.

#### Energy metabolism (organic acids) (LC-MS/MS)

For the quantitative analysis of energy metabolism intermediates (glycolysis, citrate cycle, pentose phosphate pathway, urea cycle), a hydrophilic interaction liquid chromatography (HILIC)-ESI-MS/MS method in a highly selective negative MRM detection mode was used. The MRM detection was performed using an API4000 QTrap® tandem mass spectrometry instrument (Applied Biosystems/MDS Analytical Technologies, Foster City, CA). A 20 µL sample volume (plasma) was protein-precipitated and simultaneously extracted with aqueous methanol in a 96-well plate format. Internal standards (ratio external to internal standard) and external calibration were used for highly accurate quantification. Data were quantified with Analyst 1.4.2 software (Applied Biosystems, Foster City, CA) and finally exported for statistical analysis.

### Statistics

All statistical calculations were performed using the statistics software R. Analytes that were detected in at least 15% of the samples were selected for further analyses, resulting in a list of 213 compounds/metabolites along with 28 known compound/metabolite sums and ratios (given in Table S1). Analysis of variance and covariance followed up with Tukey's ‘Honest Significant Difference’ were used to assess potential bias in the experimental design and changes associated with clinical parameters. For metabolite measurement, the gls function in the package nlme was used to compute the linear models, specifying within-treatment group heteroscedasticity structure and default parameters otherwise. Metabolite significance levels are presented in the form of q values after adjustment by the method described in Benjamini and Hochberg [47] With the exception of fold change (FC) determination, all statistical analyses were performed on pre-processed, log-transformed data. Fold changes were computed from the median ratio of original concentrations between two time points and are presented as changes compared to the control group (positive values) or to the treated group (negative values).

Due to a combination of the metabolic pathway dynamism, complex sample molecular interactions and overall efficiency of the analytical protocol, the replacement of missing data by means of a multivariate algorithm is preferred to a naive imputation by a pre-specified value like, for instance, zero. For the multivariate analysis only, the missing metabolite concentrations were replaced by a linear combination of the six most correlated analytes according to Kim et al. [48] with the method available in the R package pcaMethods.

Heat-maps consist of explicit representations of concentration ratios between two time points, where metabolites (row) and samples (columns) are reordered in an unsupervised fashion. Column-wise, samples were reordered following complete hierarchical cluster agglomeration, and, row-wise, metabolites were clustered using k means, where k is set to 5. Concentration ratios are log10-transformed and color-coded following the left corner diagram: red (resp. blue)-colored cells indicate higher (resp. lower) concentrations in the sample from the second time point.

Partial Least Square (PLS) regression [49] was carried out with the R package pls. Data were mean centered and scaled to unit variance, and the optimal number of components was determined by leaving one out validation. The PLS model's predictive abilities are assessed using 10-fold cross-validation, where 90% of the samples are used to build the regression model and the remaining 10% are employed to calculate the predictive errors [50]. PLS models are reported [49]in term of the coefficients of determination calculated from the fitted values on the overall dataset (R^2^) and from the predicted values (Q^2^), in addition to the root mean square of the predicted error (RMSE). The significance of the findings are further assessed by subjecting the previous procedure to permutation testing (n  = 5000). The resulting empirical p value corresponds to the proportion of models with higher RMSE than the PLS model built on the true data. Variable importance values in the projection (VIP) are computed according to [51]. To evaluate the stability of the variable importance score under resampling, VIP values are calculated at each cross validation step and are presented as the median and the 10% and 90% quantiles.

## Supporting Information

Table S1 List of analyzed metabolites.
(0.07 MB PDF)
