# Supplementary material for: Metabolomic Analyses of Plasma Reveals New Insights into Asphyxia and Resuscitation in Pigs
Source: PLoS One. 2010 Mar 9;5(3):e9606. doi: 10.1371/journal.pone.0009606 (PMC2834759; doi:10.1371/journal.pone.0009606)
Supplement: Table S1 — List of analyzed metabolites. (0.07 MB PDF) [file pone.0009606.s001.pdf]

Supplement:

Table 1

List of analyzed metabolites

Acylcarnitines (41)		
C0	Carnitine	quantitative
C2	Acetylcarnitine	quantitative
C3	Propionylcarnitine	quantitative
C3:1	Propenoylcarnitine	semi-quantitative
C3-OH	Hydroxypropionylcarnitine	semi-quantitative
C4	Butyrylcarnitine / Isobutyrylcarnitine	quantitative
C4:1	Butenoylcarnitine	semi-quantitative
C4-OH (C3-DC)	Hydroxybutyrylcarnitine (Malonylcarnitine)	semi-quantitative
C5	Valerylcarnitine / Isovalerylcarnitine / Methylbutyrylcarnitine	quantitative
C5:1	Tiglylcarnitine / Methylcrotonylcarnitine	semi-quantitative
C5:1-DC	Glutaconylcarnitine / Mesaconylcarnitine	semi-quantitative
C5-DC (C6-OH)	Glutarylcarnitine (Hydroxyhexanoylcarnitine)	semi-quantitative
C5-M-DC	Methylglutarylcarnitine	semi-quantitative
C5-OH (C3-DC-M)	Hydroxyvaleryl- / -isovaleryl- / -methylbutyrylcarnitine (Methylmalonylcarnitine)	semi-quantitative
C6 (C4:1-DC)	Hexanoylcarnitine (Fumarylcarnitine)	semi-quantitative
C6:1	Hexenoylcarnitine	semi-quantitative
C7-DC	Pimelylcarnitine	semi-quantitative
C8	Octanoylcarnitine	quantitative
C8:1	Octenoylcarnitine	semi-quantitative
C9	Nonanoylcarnitine	semi-quantitative
C10	Decanoylcarnitine	quantitative
C10:1	Decenoylcarnitine	semi-quantitative
C10:2	Decadienylcarnitine	semi-quantitative
C12	Dodecanoylcarnitine	quantitative
C12:1	Dodecenoylcarnitine	semi-quantitative
C12-DC	Dodecanedioylcarnitine	semi-quantitative
C14	Tetradecanoylcarnitine	quantitative
C14:1	Tetradecenoylcarnitine	semi-quantitative
C14:1-OH	Hydroxytetradecenoylcarnitine	semi-quantitative
C14:2	Tetradecadienylcarnitine	semi-quantitative
C14:2-OH	Hydroxytetradecadienylcarnitine	semi-quantitative
C16	Hexadecanoylcarnitine	quantitative
C16:1	Hexadecenoylcarnitine	semi-quantitative
C16:1-OH	Hydroxyhexadecenoylcarnitine	semi-quantitative
C16:2	Hexadecadienylcarnitine	semi-quantitative
C16:2-OH	Hydroxyhexadecadienylcarnitine	semi-quantitative
C16-OH	Hydroxyhexadecanoylcarnitine	semi-quantitative
C18	Octadecanoylcarnitine	quantitative
C18:1	Octadecenoylcarnitine	semi-quantitative
C18:1-OH	Hydroxyoctadecenoylcarnitine	semi-quantitative
C18:2	Octadecadienylcarnitine	semi-quantitative

Amino acids (21)	
Alanine	quantitative
Arginine	quantitative
Asparagine	quantitative
Aspartic acid	quantitative
Citrulline	quantitative
Glutamine	quantitative
Glutamic acid	quantitative
Glycine	quantitative
Histidine	quantitative
Isoleucine	quantitative
Leucine	quantitative
Lysine	semi-quantitative
Methionine	quantitative
Ornithine	quantitative
Phenylalanine	quantitative
Proline	quantitative
Serine	quantitative
Threonine	quantitative
Tryptophan	quantitative
Tyrosine	quantitative
Valine	quantitative

Biogenic Amines and Polyamines (14)	
Asymmetric dimethylarginine	quantitative
Symmetric dimethylarginine	quantitative
Total dimethylarginine	quantitative
Histamine	quantitative
Methionine-sulfoxide	quantitative
Kynurenine	quantitative
Hydroxykynurenine	quantitative
Putrescine	quantitative
Spermidine	semi-quantitative
Spermine	semi-quantitative
Serotonin	semi-quantitative
Phenylethylamine	quantitative
Nitrotyrosine	quantitative
Creatinine	quantitative

Bile acids (17)	
Cholic acid	quantitative
Chenodeoxycholic acid	quantitative
Deoxycholic acid	quantitative
Glycocholic acid	quantitative
Glycochenodeoxycholic acid	quantitative
Glycodeoxycholic acid	quantitative
Glycolithocholic acid	quantitative
Glycolithocholic acid sulfate	quantitative
Glycoursodeoxycholic acid	quantitative
Lithocholic acid	quantitative
Taurocholic acid	quantitative
Taurochenodeoxycholic acid	quantitative
Taurodeoxycholic acid	quantitative
Taurolithocholic acid	quantitative
Taurolithocholic acid sulfate	quantitative
Tauroursodeoxycholic acid	quantitative
Ursodeoxycholic acid	quantitative

Eicosanoids and other Oxidation Products of Polyunsaturated Fatty acids (PUFA’s)* (17)	
Leukotriene B4	quantitative
Thromboxane B2	quantitative
Leukotriene D4	quantitative
Prostaglandin E2	quantitative
8-iso-Prostaglandin F2α	quantitative
Prostaglandin F2α	quantitative
6-keto-Prostaglandin F1α	quantitative
Prostaglandin D2	quantitative
9S-HODE	quantitative
13S-HODE	quantitative
14(15)-EpETE	quantitative
12S-HETE	quantitative
15S-HETE	quantitative
15S-HpETE	quantitative
5S-HpETE	quantitative
Arachidonic acid	quantitative
Docosahexaenoic acid	quantitative

Energy Metabolism (17)	
3-Phosphoglycerate	semi-quantitative
α-Ketoglutaric acid	quantitative
Adenosine-5'-monophosphate	semi-quantitative
Arginine	quantitative
Aspartate	quantitative
3'-5'-cyclic Adenosine monophosphate	semi-quantitative
Dihydroxyacetonephosphate + 3-Phosphoglyceraldehyde	semi-quantitative

Fumarate	quantitative
Glutamate	quantitative
Hexose (e.g. Glucose)	quantitative
Hexosephosphate (e.g. Glucose-1-phosphate + Glucose-6-phosphate + Fructose-6-phosphate)	quantitative
Lactate	quantitative
Pentosephosphate (e.g. Ribose-5-phosphate + Ribulose-5-phosphate)	semi-quantitative
Phosphoenolpyruvate	semi-quantitative
Pyruvate + Oxaloacetate	quantitative
Succinate	quantitative
Tetrosephosphate (e.g. Erythrose-4-phosphate)	semi-quantitative

Glycerophospholipids* (92)			
PC aa C24:0	semi-quantitative	PC ae C34:3	semi-quantitative
PC aa C26:0	semi-quantitative	PC ae C36:0	semi-quantitative
PC aa C28:1	semi-quantitative	PC ae C36:1	semi-quantitative
PC aa C30:0	semi-quantitative	PC ae C36:2	semi-quantitative
PC aa C30:2	semi-quantitative	PC ae C36:3	semi-quantitative
PC aa C32:0	semi-quantitative	PC ae C36:4	semi-quantitative
PC aa C32:1	semi-quantitative	PC ae C36:5	semi-quantitative
PC aa C32:2	semi-quantitative	PC ae C38:0	semi-quantitative
PC aa C32:3	semi-quantitative	PC ae C38:1	semi-quantitative
PC aa C34:1	semi-quantitative	PC ae C38:2	semi-quantitative
PC aa C34:2	semi-quantitative	PC ae C38:3	semi-quantitative
PC aa C34:3	semi-quantitative	PC ae C38:4	semi-quantitative
PC aa C34:4	semi-quantitative	PC ae C38:5	semi-quantitative
PC aa C36:0	semi-quantitative	PC ae C38:6	semi-quantitative
PC aa C36:1	semi-quantitative	PC ae C40:0	semi-quantitative
PC aa C36:2	semi-quantitative	PC ae C40:1	semi-quantitative
PC aa C36:3	semi-quantitative	PC ae C40:2	semi-quantitative
PC aa C36:4	semi-quantitative	PC ae C40:3	semi-quantitative
PC aa C36:5	semi-quantitative	PC ae C40:4	semi-quantitative
PC aa C36:6	semi-quantitative	PC ae C40:5	semi-quantitative
PC aa C38:0	semi-quantitative	PC ae C40:6	semi-quantitative
PC aa C38:1	semi-quantitative	PC ae C42:0	semi-quantitative
PC aa C38:3	semi-quantitative	PC ae C42:1	semi-quantitative
PC aa C38:4	semi-quantitative	PC ae C42:2	semi-quantitative
PC aa C38:5	semi-quantitative	PC ae C42:3	semi-quantitative
PC aa C38:6	semi-quantitative	PC ae C42:4	semi-quantitative
PC aa C40:1	semi-quantitative	PC ae C42:5	semi-quantitative
PC aa C40:2	semi-quantitative	PC ae C44:3	semi-quantitative
PC aa C40:3	semi-quantitative	PC ae C44:4	semi-quantitative
PC aa C40:4	semi-quantitative	PC ae C44:5	semi-quantitative
PC aa C40:5	semi-quantitative	PC ae C44:6	semi-quantitative
PC aa C40:6	semi-quantitative	lysoPC a C6:0	semi-quantitative
PC aa C42:0	semi-quantitative	lysoPC a C14:0	semi-quantitative
PC aa C42:1	semi-quantitative	lysoPC a C16:0	semi-quantitative
PC aa C42:2	semi-quantitative	lysoPC a C16:1	semi-quantitative

PC aa C42:4	semi-quantitative	lysoPC a C17:0	semi-quantitative
PC aa C42:5	semi-quantitative	lysoPC a C18:0	semi-quantitative
PC aa C42:6	semi-quantitative	lysoPC a C18:1	semi-quantitative
PC ae C30:0	semi-quantitative	lysoPC a C18:2	semi-quantitative
PC ae C30:1	semi-quantitative	lysoPC a C20:3	semi-quantitative
PC ae C30:2	semi-quantitative	lysoPC a C20:4	semi-quantitative
PC ae C32:1	semi-quantitative	lysoPC a C24:0	semi-quantitative
PC ae C32:2	semi-quantitative	lysoPC a C26:0	semi-quantitative
PC ae C34:0	semi-quantitative	lysoPC a C26:1	semi-quantitative
PC ae C34:1	semi-quantitative	lysoPC a C28:0	semi-quantitative
PC ae C34:2	semi-quantitative	lysoPC a C28:1	semi-quantitative

**Oxysterols (16)**	
22-R-Hydroxycholesterol	quantitative
24-S-Hydroxycholesterol	quantitative
25-Hydroxycholesterol	quantitative
27-Hydroxycholesterol	quantitative
22S-Hydroxycholesterol	quantitative
24,25-Epoxycholesterol	quantitative
7α-Hydroxycholesterol	quantitative
7-Ketocholesterol	quantitative
5ß,6ß-Epoxycholesterol	quantitative
5α,6α-Epoxycholesterol	quantitative
4ß-Hydroxycholesterol	quantitative
Desmosterol	quantitative
7-Dehydrocholesterol	quantitative
Cholestenone	quantitative
Lanosterol	semi-quantitative
24-Dihydrolanosterol	quantitative

**Sphingolipids (Sphingomyelins)* (15)**	
SM (OH) C14:1	semi-quantitative
SM C16:0	semi-quantitative
SM C16:1	semi-quantitative
SM (OH) C16:1	semi-quantitative
SM C18:0	semi-quantitative
SM C18:1	semi-quantitative
SM C20:2	semi-quantitative
SM C22:3	semi-quantitative
SM (OH) C22:1	semi-quantitative
SM (OH) C22:2	semi-quantitative
SM C24:0	semi-quantitative
SM C24:1	semi-quantitative
SM (OH) C24:1	semi-quantitative
SM C26:0	semi-quantitative
SM C26:1	semi-quantitative

*) validated analytical method (FDA guidance for industry)

Analysed ratio`s

Definition	Numerator	Denominator
Orn/Cit	Ornithine	Citrulline
Orn/Arg	Ornithine	Arginine
Cit/Arg	Citrulline	Arginine
Glu/Gln	Glutamate	Glutamine
Asp/Asn	Aspartic acid	Asparagine
Ala/Lys	Alanine	Lysine
Phe/Tyr	Phenylalanine	Tyrosine
Serotonin/Trp	Serotonin	Tryptophane
Kyn/Trp	Kynurenine	Tryptophane
Kyn/OHKyn	Kynurenine	Hydroykynurenine
Putrescine/Orn	Putrescine	Ornithine
Spermine/Spermidine	Spermine	Spermidine
SDMA/ADMA	Symmetric dimethylarginine	Asymmetric dimethylarginine
Met-SO/Met	Methionine-Sulfoxide	Methionine
Ala/BCAA	Alanine	G.M. of Valine, Isoleucine and Leucine
Gly/BCAA	Glycine	G.M. of Valine, Isoleucine and Leucine
SumLyso	G.M. of 15 LysoPC	
SumPC+Lyso	G.M. of 92 LysoPC and PC	
SumPC	G.M. of 77 PC	
SumSM	G.M. of 15 SM	
SumSMOH/SumSM	G.M. of 5 SM(OH)	G.M. of 10 SM
C16+C18/C0	G.M. of palmitoylcarnitine and stearylcarnitine	Carnitine (free)
SumMUFA	G.M. of 26 mono-unsaturated lipids	
SumPUFA	G.M. of 82 poly-unsaturated lipids	
SumSFA	G.M. of 25 saturated lipids	
PUFA/SFA	G.M. of 82 poly-unsaturated lipids	G.M. of 25 saturated lipids
PUFA/MUFA	G.M. of 82 poly-unsaturated lipids	G.M. of 26 mono-unsaturated lipids
MUFA/SFA	G.M. of 26 mono-unsaturated lipids	G.M. of 25 saturated lipids
